# Smokers who have not tried alternative nicotine products: a 2019 survey of adults in Great Britain

**DOI:** 10.1186/s12954-020-00391-2

**Published:** 2020-07-14

**Authors:** Erikas Simonavicius, Ann McNeill, Hazel Cheeseman, Deborah Arnott, Leonie S. Brose

**Affiliations:** 1 Department of Addictions, Institute of Psychiatry, Psychology and Neuroscience, King’s College London, 4 Windsor Walk, London, SE5 8BB UK; 2 Action on Smoking and Health, London, UK

**Keywords:** Nicotine, Smoking, E-cigarettes, Nicotine replacement therapy, Harm reduction

## Abstract

**Aims:**

Switching from smoking to using nicotine replacement therapy (NRT), electronic cigarettes (e-cigarettes) or heated tobacco products can reduce tobacco-related health risks. However, not all smokers in Great Britain have tried these products. This study aimed to identify and describe smokers who have never tried alternative nicotine products.

**Methods:**

We analysed cross-sectional survey data of smokers (*n* = 1777) from a representative adult sample from Great Britain. The online survey was run in March 2019. The proportion of smokers who had never used alternative nicotine products was measured. A multivariate logistic regression assessed the association between never having used alternative nicotine products and sociodemographic and smoking characteristics and motivation to stop smoking.

**Results:**

One in four smokers (27.8%, 95% CI 25.8–29.9%) had never tried NRT, e-cigarettes or heated tobacco products. These smokers were more commonly from Black and Minority than White ethnic groups (AOR = 1.55; 95% CI 1.02–2.31), were more likely to smoke up to 10 versus more cigarettes per day (AOR = 1.52; 95% CI 1.14–2.03) and to report low versus moderate or high motivation to stop smoking (AOR = 1.79; 95% CI 1.20–2.74).

**Conclusion:**

Light smokers, those unmotivated to stop and smokers from Black and Minority ethnic groups are less likely to have ever tried alternative nicotine products. Different approaches are needed to facilitate harm reduction and smoking cessation among these groups of smokers.

## Introduction

Nicotine-containing products have a continuum of health risks, and tobacco smoking occupies the most harmful end of this continuum [1]. While stopping smoking is the best way to avert health risks, switching from smoking to less harmful nicotine products is an alternative for smokers who are not ready to stop [2]. The most used alternative nicotine products in the United Kingdom (UK) are electronic cigarettes (e-cigarettes) and nicotine replacement therapy (NRT); heated tobacco products (HTP) were also introduced to the market in 2016.

Since the late 1970s when nicotine gum was released as a smoking cessation aid for smokers, multiple trials have shown that NRT is effective for reducing and stopping smoking in clinical [3] but less so in real-world settings [4]. In contrast, e-cigarettes’ effectiveness for smoking cessation has been shown in clinical and real-world studies in the UK [5, 6] and they are used by a third of smokers trying to quit [7]. Around one in five current smokers use e-cigarettes concurrently, fewer—around 4–8%—concurrently use NRT [7].

E-cigarettes and NRT help to stop smoking, but it is debatable to what extent they help to reduce tobacco-related harm while smoking. Concurrent use of NRT or e-cigarettes while smoking is unlikely to substantially reduce exposure to harmful toxicants [8]. However, getting nicotine from alternative products helps smokers to reduce the number of cigarettes smoked [9] and facilitates quit attempts [10]. Concurrent use can also signal smokers’ intention to change. Compared with smokers not using alternative nicotine products, NRT users have made more recent attempts to quit smoking [4], and smokers who use e-cigarettes are more motivated to stop smoking [11].

Additionally, in 2017, 4.1% of smokers in Great Britain had tried novel HTP [12]. HTPs’ potential to reduce tobacco-related harm is promising but contested [13], particularly as they are not intended for smoking cessation [14].

This study aimed to identify smokers in Great Britain who have never used alternative nicotine products. Research questions were:

1. What proportion of smokers in Great Britain has never used e-cigarettes, NRT or HTP?
2. What smokers’ characteristics are associated with never having used any alternative nicotine product?

## Methods

This is a secondary analysis of cross-sectional data from an online survey conducted in March 2019 by the market research company YouGov and commissioned by the charity Action on Smoking and Health. To achieve a representation of the adult population of Great Britain, participants were invited in line with quotas for age, gender, social grade, newspaper readership, ethnicity and region. After the survey, data were weighted by participants’ age, gender, social class, region and education. YouGov derives targets for weights from the census, large-scale probability surveys, the results of the 2016 referendum and 2017 general election and population estimates from the Office for National Statistics. The survey sample included adults (aged 18 and over) in Great Britain (*N* = 12393) of whom only current smokers (*n =* 1777, 14.3%) were analysed. Analysis of fully anonymised data did not require ethical approval. The research questions, analysis plan and a full description of measures were pre-registered (osf.io/rywg4).

Information about participants’ gender (male; female), age (18–24, 25–34, 35–44, 45–54, 55+), ethnicity (White; Black and Minority ethnic (BME)) groups [15], highest attained education (low, medium, high) and socioeconomic status (ABC1, C2DE) was collected (Additional file 1).

Participants reported their smoking status (non-daily, daily), motivation to stop smoking (MTSS; low; moderate; strong) [16] and the heaviness of smoking index (HSI; low to moderate, 0–3; high, 4–6) [17], which consists of time to first cigarette (TTFC; within 5 min, 6–30 min, 31–60 min, after 60 min, don’t know) [18] and cigarettes smoked per day (CPD 1–10, 11–20, 21–30, 31+) [19]. Ever use of alternative nicotine products (e-cigarettes, NRT and/or HTP) was a combined measure defined by responses to questions about each of the products separately (never tried, tried or used; Additional file 1).

Descriptive statistics summarised and compared sociodemographic and smoking characteristics between those who had never tried and ever tried any alternative nicotine product. Differences were assessed using *χ*^2^ and Cramer’s *V* statistics [20]. For contingency tables larger than 2 × 2, adjusted residuals greater than |2.58|—which corresponds to a significance level of *α* = 0.01—were used to identify cells contributing to the differences between groups [21].

For research question one, we estimated with 95% confidence intervals what part of the adult smoking population had never tried any alternative nicotine product. For research question two, we used unweighted data [22] and designed a complete-case multivariate logistic regression model with never use of any alternative nicotine product as an outcome variable regressed onto smokers’ characteristics. To optimise analysis, we excluded education (multicollinearity with SES) and HSI (missing data and multicollinearity with CPD and TTFC) and recoded age (18–34, 35–54, 55+), CPD (1–10, 11+) and TTFC (within 5 min, 6 to 60 min, after 60 min/don’t know) into fewer categories.

In a post hoc analysis, we separated ethnicity into ‘White’, ‘Asian’, ‘Black’ and ‘Mixed & Other’ groups [15] and ran logistic regressions predicting ever use of alternative nicotine products within each group.

## Results

Out of 1777 smokers, 37.0% (95% CI 34.8–39.2%) had never tried e-cigarettes, 63.5% (95% CI 61.3–65.7%) had never tried NRT and 95.1% (95% CI 94.0–96.0%) had never tried HTP. Combined, 27.8% (95% CI 25.8–29.9%) had never tried any of the alternative nicotine products (Table 1). A third (32.8%) has ever tried e-cigarettes only; 26.6% e-cigarettes and NRT; 8.0% NRT only; 2.3% e-cigarettes and HTP; 1.4% e-cigarettes, NRT and HTP; 0.7% HTP only; and 0.6% NRT and HTP.

**Table 1:** Sample characteristics in row percentage and counts by use of alternative nicotine products (n = 1777)

Variables	Total by variables, % (*n*)	Use of alternative nicotine products		Comparison
		Never tried	Tried or used	
		27.9% (496)	72.1% (1281)	
Gender, % (*n*)				
Male	45.5 (808)	30.1 (243)	69.9 (565)	*χ*^2^ (1) = 3.4, *p* = .064; *V* = .044
Female	54.5 (969)	26.1 (253)	73.9 (716)	
Age, % (*n*)				
18–24	14.1 (250)	34.8 (87)	65.2 (163)	*χ*^2^ (4) = 9.0, *p* = .060; *V* = .071
25–34	15.7 (279)	29.7 (83)	70.3 (196)	
35–44	16.6 (295)	24.7 (73)	75.3 (222)	
45–54	24.1 (429)	25.6 (110)	74.4 (319)	
55+	29.5 (524)	27.3 (143)	72.7 (381)	
Ethnicity, % (*n*)				
White	91.9 (1632)	27.1 (443)	72.9 (1189)	**χ**^**2**^**(1) = 6.4, p = .01; V = .060**
BME	8.0 (143)	37.1 (53)	62.9 (90)	
Missing*	0.1 (2)	(0)	(2)	
SES, % (*n*)				
ABC1	47.4 (843)	30.1 (254)	69.9 (589)	**χ**^**2**^**(1) = 3.9, p = .048; V = .047**
C2DE	52.6 (934)	25.9 (242)	74.1 (692)	
Education, % (*n*)				
Low	31.7 (564)	28.0 (158)	72.0 (406)	*χ*^2^ (2) = 0.9, *p* = .65; *V* = .022
Medium	44.9 (797)	28.7 (229)	71.3 (568)	
High	23.4 (416)	26.2 (109)	73.8 (307)	
Smoking, % (*n*)				
Non-daily	29.2 (518)	**36.3 (188)**	**63.7 (330)**	**χ**^**2**^**(1) = 25.5, p < .001; V = .120**
Daily	70.8 (1259)	**24.5 (308)**	**75.5 (951)**	
MTSS, % (*n*)				
Low	50.2 (892)	**33.0 (294)**	**67.0 (598)**	**χ**^**2**^**(2) = 24.2, p < .001; V = .117**
Moderate	38.8 (689)	**23.8 (164)**	**76.2 (525)**	
Strong	11.0 (196)	**19.4 (38)**	**80.6 (158)**	
CPD, % (*n*)				
1–10	46.3 (822)	**30.2 (248)**	**69.8 (574)**	**χ**^**2**^**(3) = 23.8, p < .001; V = .122**
11–20	32.1 (570)	**19.8 (113)**	**80.2 (457)**	
21–30	9.0 (160)	18.8 (30)	81.2 (130)	
31+	2.5 (45)	20.0 (9)	80.0 (36)	
Missing*	10.1 (180)	53.3 (96)	46.7 (84)	
TTFC, % (*n*)				
Within 5 min	16.4 (292)	20.5 (60)	79.5 (232)	**χ**^**2**^**(4) = 75.3, p < .001; V = .206**
6 to 30 min	34.7 (617)	**21.4 (132)**	**78.6 (485)**	
31 to 60 min	12.5 (222)	25.2 (56)	74.8 (166)	
After 60 min	24.9 (442)	**33.3 (147)**	**66.7 (295)**	
Don’t know	11.5 (204)	**49.5 (101)**	**50.5 (103)**	
HSI, % (*n*)				
Low to moderate (0–3)	66.5 (1182)	26.1 (308)	73.9 (874)	**χ**^**2**^**(1) = 6.7, p = .010; V = .067**
High (4–6)	18.3 (325)	19.1 (62)	80.9 (263)	
Missing*	15.2 (270)	46.7 (126)	53.3 (144)	

*Missing data are not included in *χ*^2^ tests

Proportion cells in bold are associated with adjusted residuals greater than ± 2.58 (*α* = 0.01)

*BME* Black and Minority ethnic group, *SES* socioeconomic status, *MTSS* motivation to stop smoking, *CPD* cigarettes smoked per day, *TTFC* time to the first cigarette, *HSI* heaviness of smoking index, a combination of CPD and TTFC [19]

In *χ*^*2*^ comparisons, smokers’ gender, age and education were not associated with alternative nicotine products use. Being from a BME group, higher socioeconomic status, non-daily smoking, longer time until the first cigarette, smoking up to 10 cigarettes per day, having low dependence and low motivation to stop smoking were all associated with never use of alternative nicotine products (Table 1).

The post hoc analysis indicated that smokers from Asian ethnic groups were more likely to have never tried alternative nicotine products (OR = 2.80; 95% CI 1.42–5.51; Additional file 1).

In adjusted analysis, smokers from BME groups (AOR = 1.55; 95% CI 1.02–2.31), those with low motivation to stop (AOR = 1.79; 95% CI 1.20–2.74) and those smoking up to 10 cigarettes per day (AOR = 1.52; 95% CI 1.14–2.03) were more likely to have never used alternative nicotine products (Table 2).

**Table 2:** Multivariate logistic regression model predicting never use of alternative nicotine products (*n* = 1595)

Variable	Odds Ratio	95% confidence intervals	*p*
Intercept	**0.17**	**0.10–0.29**	**< .001**
Gender			
Male	Ref		
Female	0.87	0.69–1.10	.25
Age			
18–34	0.94	0.68–1.29	.70
35–54	0.93	0.70–1.23	.59
55+	Ref		
Ethnicity			
White	Ref		
BME	**1.55**	**1.02–2.31**	**.035**
Socioeconomic status			
ABC1	1.01	0.80–1.28	.93
C2DE	Ref		
Smoking			
Non-daily	1.14	0.83–1.55	.41
Daily	Ref		
MTSS			
Low	**1.79**	**1.20–2.74**	**.005**
Moderate	1.28	0.85–1.97	.25
Strong	Ref		
CPD			
1–10	**1.52**	**1.14–2.03**	**.004**
11+	Ref		
TTFC			
Within 5 min	Ref		
6 to 60 min	1.00	0.72–1.42	.98
After 60 min/DK	1.31	0.87–1.99	.21

*BME* Black and Minority ethnic group, *MTSS* motivation to stop smoking, *CPD* cigarettes smoked per day, *TTFC* time to the first cigarette

## Discussion

Among smokers from Great Britain, over a quarter (27.8%) have never tried NRT, e-cigarettes or HTP. These smokers were more commonly from BME groups, were smoking fewer cigarettes per day and reported low motivation to stop smoking.

A few limitations are relevant to study results. Data were cross-sectional, collected online in English only and self-reported. Measures of smoking status, motivation to stop smoking and cigarettes smoked per day fluctuate [23] but were measured only at the time of the survey. Also, non-daily smokers were asked about cigarettes smoked per day, which could have biassed their CPD measure. Participants’ recall of alternative nicotine products use could have been biassed: for instance, new HTP might have been confused with e-cigarettes [12]. Nevertheless, the validity of self-reported ever use of NRT and e-cigarettes—products that most smokers are aware of [24]—should be similar to the acceptable level of validity of self-reported smoking status [25].

Three out of four participants have tried at least one alternative nicotine product. The proportion of smokers who have tried HTP was 4.9%—similar to the 4.1% from the same survey in 2017 [12]. NRT has been on the market for around 50 years and e-cigarettes only for a decade, but substantially fewer smokers have ever tried NRT than e-cigarettes. Vaping mimics behavioural, social and psychological aspects of smoking better than NRT use [26], which might explain current smokers’ preference for the newer product.

Alternative nicotine products can help to facilitate changes in smoking behaviour, but it does not imply that all participants who had tried them did so to reduce or stop smoking. Smokers try and use NRT for reasons other than reducing or stopping smoking [27], and many try e-cigarettes out of curiosity [11]. Likewise, never use of alternative nicotine products does not mean smokers are not trying to stop smoking: they might instead choose behavioural and/or medicinal support, or no support at all. Nevertheless, smokers who use alternative nicotine products have been shown to be more motivated to stop and have higher chances to stop smoking [28].

Smokers who had and had not tried alternative nicotine products did not differ in gender, age or socioeconomic status, which confirms that e-cigarettes [29] and NRT [30] are equally used by smokers from different socioeconomic backgrounds. However, smokers from BME groups were less likely to have ever tried alternative nicotine products than smokers from White ethnic groups. The post hoc analysis suggested that the difference may be due to low rates of trial in Asian smokers. More prevalent use of smokeless tobacco in South Asian groups [31] could account for the difference, but we did not collect data on this and sample sizes for different ethnicities were too small for firm conclusions. Our study is the first to highlight the disparity between White and BME smokers in Great Britain, but similar findings are common in studies from the United States (US). In the US, Black and Hispanic smokers are less likely to have ever used NRT [32] and e-cigarettes [33] than White smokers, and the differences have been attributed to more positive attitudes towards smoking [33], poorer awareness of [34] and knowledge about alternative nicotine products [35] among BME smokers, possibly due to how they are marketed [36]. Ethnicity is associated with different smoking and cessation behaviours [37], however, it is rarely accounted for in UK research on smoking and nicotine use.

Tobacco harm reduction enables smokers who do not want to or cannot stop smoking to reduce smoking-related health risks without completely ceasing to use nicotine [1]. Some smokers are reluctant to try harm reduction because they overestimate health risks associated with nicotine or e-cigarette use [38, 39]. Our findings suggest that harm reduction might also be less attractive to light smokers and those with low motivation to stop smoking. Non-daily and light smokers often do not crave nicotine or experience withdrawal [40], accordingly, they may not perceive the need for alternative nicotine products and/or feel the investment worthwhile. Similarly, smokers who enjoy smoking and do not want to quit might not see the need to try alternative products. These findings are concerning, since during the last decade smokers in England have become less dependent and less likely to try quitting [41]. Light smokers, whose numbers have been increasing in the UK and worldwide [42], struggle to stop smoking as much as heavier smokers [43] but receive less support from healthcare specialists [44]. According to our findings, they are also less likely to self-select harm reduction, thus the question remains as to how to approach and support light or unmotivated smokers to stop smoking.

This is the first study to identify lower ever use of alternative nicotine products among smokers from BME groups in Great Britain. Future research should explore reasons for this difference, and smokers’ ethnic background should not be neglected when investigating disparities in tobacco harm reduction in Great Britain.

## Conclusions

Among adult smokers from Great Britain, over a quarter has never tried NRT, e-cigarettes or heated tobacco products. Never use of alternative nicotine products was more common among smokers from BME groups, those with low motivation to stop smoking and those smoking fewer cigarettes per day. Switching from smoking to alternative nicotine sources might not be appealing or accessible to these smokers, thus new approaches to reduce the harmfulness of their smoking may be needed.

## Supplementary information

**Additional file 1.** Additional file for the study

## Data Availability

The data that support the findings of this study are available from the charity Action on Smoking and Health upon reasonable request.

## Abbreviations

AOR: Adjusted odds ratio
BME: Black and minority ethnic
CI: Confidence interval
CPD: Cigarettes smoked per day
e-cigarette: Electronic cigarette
HSI: Heaviness of smoking index
HTP: Heated tobacco products
MTSS: Motivation to stop smoking
NRT: Nicotine replacement therapy
OR: Odds ratio
SES: Socioeconomic status
TTFC: Time to first cigarette
