# Supplementary material for: Smokers who have not tried alternative nicotine products: a 2019 survey of adults in Great Britain
Source: Harm Reduct J. 2020 Jul 14;17:46. doi: 10.1186/s12954-020-00391-2 (PMC7362479; doi:10.1186/s12954-020-00391-2)
Supplement: Supplementary file 1 — Additional file 1. Additional file for the study [file 12954_2020_391_MOESM1_ESM.docx]

*Additional file for the study*

**Smokers who have not tried alternative nicotine products: a 2019 survey of adults in Great Britain**

*Erikas Simonavicius^1^, Ann McNeill^1^, Hazel Cheeseman^2^, Deborah Arnott^2^, Leonie S Brose^1^*

^1^ Department of Addictions, Institute of Psychiatry, Psychology and Neuroscience, King’s College London, London, United Kingdom

^2^ Action on Smoking and Health, London, United Kingdom

**Correspondence to**Erikas Simonavicius, Department of Addictions, Institute of Psychiatry, Psychology and Neuroscience, King’s College London, 4 Windsor Walk, London SE5 8BB, United Kingdom.
E-mail: erikas.simonavicius@kcl.ac.uk

# Methods

## Sociodemographic measures

Participants’ *gender* (male; female), *age* (18–24, 25–34, 35–44, 45–54, 55+) and *highest attained education* (low; medium; high) were collected and provided by YouGov Plc.

In the study, *socioeconomic status* was combined into ABC1 and C2DE. These broader categories are based on the UK’s social grade categorisation introduced by the National Readership survey (1). The finer categories represent the following participants’ occupations:

- A: Higher managerial, administrative and professional
- B: Intermediate managerial, administrative and professional
- C1: Supervisory, clerical and junior managerial, administrative and professional
- C2: Skilled manual workers
- D: Semi-skilled and unskilled manual workers
- E: State pensioners, casual and lowest grade workers, unemployed with state benefits only

Originally, participants have defined their *ethnicity* with a single response to the ethnicity question which matches the official list of ethnic groups provided by the Government (2):

1. White
   1. English / Welsh / Scottish / Northern Irish / British
   2. Irish
   3. Gypsy or Irish Traveller
   4. Any other White background
2. Mixed / Multiple ethnic groups
   1. White and Black Caribbean
   2. White and Black African
   3. White and Asian
   4. Any other Mixed / Multiple ethnic background
3. Asian / Asian British
   1. Indian
   2. Pakistani
   3. Bangladeshi
   4. Chinese
   5. Any other Asian background
4. Black / African / Caribbean / Black British
   1. African
   2. Caribbean
   3. Any other Black / African / Caribbean background
5. Other ethnic group
   1. Arab
   2. Any other ethnic group

In the study, participants’ *ethnicity* was combined into ‘White’ (categories 1.1-1.4) and ‘Black and Minority ethnic’ (BME) groups (categories 2.1-5.2).

## Smoking and nicotine use characteristics

Participants’ *smoking status* was defined by ‘Smoking in this survey refers to all burnt tobacco products. It does NOT include e-cigarettes. Which of the following statements BEST applies to you?’ with response options: a) I smoke but I don’t smoke every day; b) I smoke every day; c) I used to smoke but have given up now; d) I have never smoked. Only participants who responded a) or b) were included to the study and later categorised to ‘daily’ and ‘non-daily’ smokers*.*

*Motivation to stop smoking (MTSS)* was defined by ‘Which ONE of the following best describes you?’ with response options: a) I don’t want to ever stop smoking; b) I think I should stop smoking but don’t really want to; c) I want to stop smoking but haven’t thought about when; d) I want to stop smoking and hope to soon; e) I REALLY want to stop smoking but I don’t know when I will; f) I REALLY want to stop smoking and intend to in the next 3 months; g) I REALLY want to stop smoking and intend to in the next month; h) Don’t know. Participants who responded a), b) or h) were categorised as having ‘low’ motivation, those who responded c), d) or e) were categorised as having ‘moderate’ motivation and those who responded f) or g) were categorised as having ‘high’ motivation to stop smoking.

*Time to first cigarette (TTFC)* was defined by ‘How soon after waking do you usually have your first cigarette?’ with response options: a) Within 5 minutes (4 for *HSI*); b) 6 to 30 minutes (3 for *HSI*); c) 31 to 60 minutes (2 for *HSI*); d) After 60 minutes (1 for *HSI*); e) Don’t know (missing for *HSI*).

*Cigarettes smoked per day (CPD)* were defined by “For the next question please think about both ‘ready-made’ cigarettes (i.e. in packs) and ‘hand rolled tobacco cigarettes’ (e.g. made by purchasing a pack of tobacco and rolling paper to make a cigarette). On average, how many cigarettes (both ready-made and hand-rolled) do you smoke EACH DAY?” with response options: a) 6 or fewer (1 for *HSI*); b) 7 to 10 (1 for *HSI*); c) 11 to 20 (2 for *HSI*); d) 21 to 30 (3 for *HSI*); e) 31 or more (4 for *HSI*); f) Don’t know (missing for *HSI*). Participants who responded f) “Don’t know” were treated as having missing data on *CPD* variable.

*Heaviness of smoking index (HSI)* score was calculated by adding *TTFC* and *CPD* scores. Participants’ whose *HSI* score was ≤3 were categorised as having ‘low to moderate’ dependence and whose *HSI* score was >3 were categorised as having ‘high’ dependence.

*E-cigarette use status* was defined by ‘E-cigarettes are also sometimes called vapes or vaping devices. Which of the following statements BEST applies to you?’ with response options: a) I have never heard of e-cigarettes and have never tried them; b) I have heard of e-cigarettes but have never tried them; c) I have tried e-cigarettes but do not use them (anymore); d) I have tried e-cigarettes and still use them; e) Don’t know. Participants who responded a), b) or e) were categorised as ‘never tried’ and those who responded c) or d) were categorised as ‘tried or used’.

*NRT use status* was defined by ‘Which of the following statements BEST applies to your use of Nicotine Replacement Therapy (NRT) such as nicotine patches and gum?’ with response options: a) I have never heard of NRT and never tried any products; b) I have heard of NRT but never tried any products; c) I have tried NRT products but do not use them (anymore); d) I have tried NRT products and still use them; e)Don’t know. Participants who responded a), b) or e) were categorised as ‘never tried’ and those who responded c) or d) were categorised as ‘tried or used’.

*HTP use status* was defined by ‘Heat not burn tobacco products, also known as heated tobacco products, use a technology whereby tobacco is being heated as opposed to being burnt, these are NOT the same as e-cigarettes. Some brands of heat not burn tobacco products include Glo and iQos Heets. Thinking about heat not burn tobacco products, which of the following statements BEST applies to you?’ with response options: a) I have never heard of heat not burn tobacco products and have never tried them; b) I have heard of heat not burn tobacco products but have never tried them; c) I have tried heat not burn tobacco products but do not use them (anymore); d) I have tried heat not burn tobacco products and still use them; e) Don’t know. Participants who responded a), b) or e) were categorised as ‘never tried’ and those who responded c) or d) were categorised as ‘tried or used’.

*Use of alternative nicotine products* was a measure combined from *e-cigarette use status, NRT use status* and *HTP use status.* Participants who have ever ‘tried or used’ at least one of these products were categorised as ‘tried or used’ and those who have never tried any of the products were categorised as ‘never tried’.

# Results

**Table S1** Bivariate logistic regressions predicting never use of alternative nicotine products within different ethnicity groups (n = 1777).

| **Variable** | ***n*** | **% used alternative nicotine products** | **Odds Ratio** | **95% confidence intervals** | ***p*** |
| --- | --- | --- | --- | --- | --- |
| **Ethnicity** |  |  |  |  |  |
| White | 1632 | 27.1 | **0.63** | **0.45 – 0.91** | **.012** |
| Asian | 35 | 51.4 | **2.80** | **1.42 – 5.51** | **.003** |
| Black | 18 | 33.3 | 1.29 | 0.45 – 3.35 | .61 |
| Mixed & Other | 90 | 32.2 | 1.24 | 0.78 – 1.94 | .35 |

# References

1. National Readership Survey. Social Grade 2020 [Available from: <http://www.nrs.co.uk/nrs-print/lifestyle-and-classification-data/social-grade/>.

2. HM Government. List of ethnic groups 2020 [Available from: <https://www.ethnicity-facts-figures.service.gov.uk/ethnic-groups>.
